# Supplementary material for: Drosophila embryos allocate lipid droplets to specific lineages to ensure punctual development and redox homeostasis
Source: PLoS Genet. 2023 Aug 14;19(8):e1010875. doi: 10.1371/journal.pgen.1010875 (PMC10449164; doi:10.1371/journal.pgen.1010875)
Supplement: S8 Table — Gene ontology scores determined by FlyEnrichr for genes which are differentially regulated in all 3 lipid droplet mutant embryos (Jabba-/-, klar-/- and dPlin2-/-). The columns from left to right show the gene ontology term, how many of our DE genes match that term out of the total, P-value which indicates if there is a significant relationship between our mutants and this GO term, Adjusted P-value which corrects P-values for multiple testing, Old P-value calculated from a previous version of FlyEnrichr, Old adjusted P-value calculated from a previous version of FlyEnrichr, z-score which is computed using a modification to Fisher’s exact test providing rank data, combined score is a combination of the p-value and z-score, and the DE genes matching the GO term. (PDF) [file pgen.1010875.s008.pdf]

Biological Process

Term	Overlap	P-value	Adjusted P-value	Old P-value	Old Adjusted P-v	Z-score	Combined Score	Genes
response to copp	2/7	0.000629503970	0.08782471169	0.001646962864	0.2328092005	-4.280103473	31.5468382	CG10505;Grx1
peptide metaboli	4/72	0.000691533162	0.08782471169	0.001833143311	0.2328092005	-2.70619047	19.69186408	GstD9;GstT4;CG7791;Psa
regulation of prim	2/15	0.003057496538	0.1728569705	0.006004592906	0.237659562	-3.267188341	18.9175394	Gabat;bwa
purine ribonucleo	1/6	0.03284541638	0.1728569705	0.04755123139	0.237659562	-5.40459602	18.46179235	w
ocellus pigment b	2/20	0.005433447565	0.1728569705	0.009975592665	0.237659562	-3.362278098	17.53489032	w;mal
ommochrome me	2/20	0.005433447565	0.1728569705	0.009975592665	0.237659562	-3.150778705	16.43188261	w;mal
tetrahydrobiopter	1/6	0.03284541638	0.1728569705	0.04755123139	0.237659562	-4.803386791	16.40809585	pr
temperature com	1/6	0.03284541638	0.1728569705	0.04755123139	0.237659562	-4.762098521	16.26705747	Ir25a
positive regulatio	1/6	0.03284541638	0.1728569705	0.04755123139	0.237659562	-4.623325675	15.79301732	GILT2
malate transmem	1/7	0.03821473511	0.1728569705	0.05416045197	0.237659562	-4.832840037	15.77697111	Bmcp
oxaloacetate trar	1/7	0.03821473511	0.1728569705	0.05416045197	0.237659562	-4.825147646	15.75185904	Bmcp
succinate transm	1/7	0.03821473511	0.1728569705	0.05416045197	0.237659562	-4.820259892	15.7359028	Bmcp
thiosulfate transp	1/7	0.03821473511	0.1728569705	0.05416045197	0.237659562	-4.814663913	15.71763453	Bmcp
pteridine metabo	1/6	0.03284541638	0.1728569705	0.04755123139	0.237659562	-4.522294405	15.44790026	pr
aminergic neurot	1/6	0.03284541638	0.1728569705	0.04755123139	0.237659562	-4.427507666	15.12411416	w
regulation of defe	1/8	0.04355451225	0.1728569705	0.0607246381	0.237659562	-4.798211302	15.03635614	GILT2
positive regulatio	1/7	0.03821473511	0.1728569705	0.05416045197	0.237659562	-4.582950047	14.96119671	rk
mitochondrial cal	1/6	0.03284541638	0.1728569705	0.04755123139	0.237659562	-4.364971148	14.91049297	MCU
positive regulatio	1/7	0.03821473511	0.1728569705	0.05416045197	0.237659562	-4.500514859	14.69208423	GILT2
response to zinc	1/6	0.03284541638	0.1728569705	0.04755123139	0.237659562	-4.11112074	14.04335442	CG10505
amine transport (	1/6	0.03284541638	0.1728569705	0.04755123139	0.237659562	-4.000371429	13.66504108	w
purine ribonucleo	1/7	0.03821473511	0.1728569705	0.05416045197	0.237659562	-4.17381554	13.62556316	Adk3
negative regulati	1/6	0.03284541638	0.1728569705	0.04755123139	0.237659562	-3.94559769	13.47793711	Fs
positive regulatio	1/8	0.04355451225	0.1728569705	0.0607246381	0.237659562	-4.295394249	13.46065724	GILT2
activation of ader	1/6	0.03284541638	0.1728569705	0.04755123139	0.237659562	-3.9004715	13.32378861	rk
de novo' pyrimidi	1/9	0.04886490887	0.177495799	0.06724409102	0.237659562	-4.377818882	13.21530325	Dhod
mitochondrial cal	1/6	0.03284541638	0.1728569705	0.04755123139	0.237659562	-3.806161848	13.00163221	MCU
monocarboxylic a	1/7	0.03821473511	0.1728569705	0.05416045197	0.237659562	-3.910209317	12.76501166	Gabat
glycerolipid catab	1/7	0.03821473511	0.1728569705	0.05416045197	0.237659562	-3.910033217	12.76443678	CG11619
cytosolic calcium	1/6	0.03284541638	0.1728569705	0.04755123139	0.237659562	-3.727983969	12.73458103	MCU
succinate transpo	1/9	0.04886490887	0.177495799	0.06724409102	0.237659562	-4.137218378	12.48900353	Bmcp
pyrimidine nucleo	1/9	0.04886490887	0.177495799	0.06724409102	0.237659562	-4.087595809	12.3392081	Dhod
neurotransmitter	2/25	0.008425684258	0.1728569705	0.01482651823	0.237659562	-2.560180692	12.22862778	CG13795;CG13796
microtubule bunc	2/22	0.006558381144	0.1728569705	0.01181395033	0.237659562	-2.422224226	12.176549	pav;l(2)41Ab
pteridine-containi	1/10	0.05414608515	0.177495799	0.07371910995	0.237659562	-4.125666936	12.03073195	pr
regulation of tran	1/8	0.04355451225	0.1728569705	0.0607246381	0.237659562	-3.82421368	11.98409891	udd
aromatic compou	2/27	0.009787248028	0.1728569705	0.0169991333	0.237659562	-2.56834605	11.88290236	w;mal
regulation of auto	1/8	0.04355451225	0.1728569705	0.0607246381	0.237659562	-3.757769775	11.77588086	SREBP
modification-depe	1/7	0.03821473511	0.1728569705	0.05416045197	0.237659562	-3.591618698	11.72496172	Ubi-p5E
response to meta	2/21	0.00598373337	0.1728569705	0.01087743378	0.237659562	-2.282399308	11.68294152	CG10505;Grx1
nucleobase-conta	1/11	0.05939820044	0.177495799	0.08014999217	0.237659562	-4.126039925	11.64983803	DNaseII
heterocycle biosy	2/29	0.01123962382	0.1728569705	0.01929861521	0.237659562	-2.590610231	11.62746156	w;mal
GTP metabolic p	1/6	0.03284541638	0.1728569705	0.04755123139	0.237659562	-3.388860448	11.57615438	mal
phosphate ion tra	1/10	0.05414608515	0.177495799	0.07371910995	0.237659562	-3.946874138	11.50935971	Bmcp
malate transport	1/10	0.05414608515	0.177495799	0.07371910995	0.237659562	-3.845435585	11.21355782	Bmcp
mitochondrial pro	1/8	0.04355451225	0.1728569705	0.0607246381	0.237659562	-3.501297147	10.97216182	CG7791
phosphate ion tra	1/11	0.05939820044	0.177495799	0.08014999217	0.237659562	-3.815144424	10.77202727	Bmcp
trehalose metabo	1/7	0.03821473511	0.1728569705	0.05416045197	0.237659562	-3.297826633	10.76586751	CG5171
organic cyclic coi	2/31	0.01278067165	0.1728569705	0.02172117615	0.237659562	-2.445687398	10.66275995	w;mal
sphingolipid cata	1/7	0.03821473511	0.1728569705	0.05416045197	0.237659562	-3.196289329	10.43439551	bwa
sodium ion transı	2/27	0.009787248028	0.1728569705	0.0169991333	0.237659562	-2.209109309	10.22083073	CG13795;CG13796
regulation of mici	1/10	0.05414608515	0.177495799	0.07371910995	0.237659562	-3.457053099	10.08100746	pav
phospholipid cata	1/8	0.04355451225	0.1728569705	0.0607246381	0.237659562	-3.1621024	9.909213006	CG11619
cellular biogenic	1/7	0.03821473511	0.1728569705	0.05416045197	0.237659562	-2.925558176	9.550584431	w
cellular response	2/31	0.01278067165	0.1728569705	0.02172117615	0.237659562	-2.161965404	9.425782765	Prx2540-1;Grx1
coenzyme metab	1/9	0.04886490887	0.177495799	0.06724409102	0.237659562	-3.12036906	9.419444822	mal
monocarboxylic a	2/34	0.01525390951	0.1728569705	0.02557769441	0.237659562	-2.243277268	9.383448112	CG4830;SREBP
mitotic spindle ele	1/10	0.05414608515	0.177495799	0.07371910995	0.237659562	-3.216769927	9.380325009	pav
actomyosin contr	1/12	0.06462141319	0.1779940862	0.086537033	0.237659562	-3.416667337	9.358967456	pav
regulation of lipid	1/12	0.06462141319	0.1779940862	0.086537033	0.237659562	-3.401764868	9.318146471	bwa
contractile ring co	1/12	0.06462141319	0.1779940862	0.086537033	0.237659562	-3.378911096	9.255545204	pav
neurotransmitter	1/8	0.04355451225	0.1728569705	0.0607246381	0.237659562	-2.924198398	9.163683249	w
protein modificati	1/10	0.05414608515	0.177495799	0.07371910995	0.237659562	-3.129250354	9.125111843	Ubi-p5E
positive regulatio	1/10	0.05414608515	0.177495799	0.07371910995	0.237659562	-3.121893293	9.103658139	udd
determination of	1/11	0.05939820044	0.177495799	0.08014999217	0.237659562	-3.215011317	9.07755664	l(2)41Ab
hydrogen peroxid	1/12	0.06462141319	0.1779940862	0.086537033	0.237659562	-3.313374226	9.076025989	Prx2540-1
cellular macromo	1/14	0.07498176072	0.1779940862	0.09918076211	0.237659562	-3.483168101	9.023183139	DNaseII
apoptotic DNA fra	1/11	0.05939820044	0.177495799	0.08014999217	0.237659562	-3.188233793	9.001950533	DNaseII
ceramide metabo	1/11	0.05939820044	0.177495799	0.08014999217	0.237659562	-3.185918512	8.995413356	bwa

tetrahydrobiopter	1/6	0.03284541638	0.1728569705	0.04755123139	0.237659562	-2.608457005	8.910340644	pr		
glycerophospholi	1/6	0.03284541638	0.1728569705	0.04755123139	0.237659562	-2.596265944	8.868696674	CG11619		
apoptotic nuclear	1/11	0.05939820044	0.177495799	0.08014999217	0.237659562	-3.1001822	8.753337622	DNaseII		
ommochrome bio	2/20	0.005433447565	0.1728569705	0.009975592665	0.237659562	-1.661632197	8.665713388	w;mal		
regulation of cellu	1/13	0.06981588103	0.1779940862	0.09288052583	0.237659562	-3.207079204	8.536904164	SREBP		
fatty acid biosynt	3/57	0.003915629681	0.1728569705	0.008164597583	0.237659562	-1.505626356	8.345354335	CG4830;SREBP;CG9458		
eye pigment bios	2/39	0.01979001567	0.1728569705	0.03256912583	0.237659562	-2.122516636	8.325736485	w;mal		
regulation of micr	1/13	0.06981588103	0.1779940862	0.09288052583	0.237659562	-3.062589252	8.152287261	pav		
regulation of lipid	1/6	0.03284541638	0.1728569705	0.04755123139	0.237659562	-2.304616199	7.872437749	SREBP		
cholesterol transp	1/14	0.07498176072	0.1779940862	0.09918076211	0.237659562	-2.964220576	7.678844186	w		
sphingolipid meta	2/38	0.01884256235	0.1728569705	0.03111649437	0.237659562	-1.932895734	7.676760241	CG9458;bwa		
protein processin	1/6	0.03284541638	0.1728569705	0.04755123139	0.237659562	-2.23747723	7.643094852	CG7791		
carboxylic acid tr	1/13	0.06981588103	0.1779940862	0.09288052583	0.237659562	-2.86937991	7.637984514	Sln		
cellular amide me	1/11	0.05939820044	0.177495799	0.08014999217	0.237659562	-2.626711359	7.416496797	CG7791		
sulfur compound	1/14	0.07498176072	0.1779940862	0.09918076211	0.237659562	-2.784849946	7.214182708	Bmcp		
eye pigment meta	1/12	0.06462141319	0.1779940862	0.086537033	0.237659562	-2.633496937	7.213699695	w		
stabilization of m	1/15	0.08011920815	0.1850025352	0.1054380314	0.2456996327	-2.842051611	7.174019369	CG42340		
membrane lipid m	1/14	0.07498176072	0.1779940862	0.09918076211	0.237659562	-2.751307207	7.127289895	bwa		
fatty acid metabo	2/42	0.02274914506	0.1728569705	0.03708324264	0.237659562	-1.858926712	7.032743054	CG4830;SREBP		
axoneme assembl	1/14	0.07498176072	0.1779940862	0.09918076211	0.237659562	-2.712602663	7.027025369	l(2)41Ab		
oligosaccharide c	1/11	0.05939820044	0.177495799	0.08014999217	0.237659562	-2.480097295	7.002533256	Mal-A4		
transmembrane t	3/75	0.00840892425	0.1728569705	0.01663347516	0.237659562	-1.455714294	6.956075039	CG10505;nAChRalpha3;w		
sulfate transport	1/16	0.08522837843	0.1882435489	0.1116526213	0.2466066592	-2.801567965	6.898639286	Bmcp		
regulation of cellu	1/18	0.09536250357	0.1968225749	0.1239549041	0.2575834274	-2.846428021	6.689304593	GluRIIC		
regulation of deve	1/13	0.06981588103	0.1779940862	0.09288052583	0.237659562	-2.502095171	6.660311554	Hr3		
C4-dicarboxylate	1/16	0.08522837843	0.1882435489	0.1116526213	0.2466066592	-2.703628668	6.657471523	Bmcp		
anesthesia-resist	1/14	0.07498176072	0.1779940862	0.09918076211	0.237659562	-2.554020148	6.616215719	MCU		
glycerophospholi	1/15	0.08011920815	0.1850025352	0.1054380314	0.2456996327	-2.560578739	6.463514383	CG11619		
regulation of deca	1/12	0.06462141319	0.1779940862	0.086537033	0.237659562	-2.337858662	6.40388454	Fs		
regulation of chiti	1/14	0.07498176072	0.1779940862	0.09918076211	0.237659562	-2.469635197	6.397615625	rk		
calcium ion impo	1/8	0.04355451225	0.1728569705	0.0607246381	0.237659562	-2.03441057	6.375317789	MCU		
mitochondrial tra	1/14	0.07498176072	0.1779940862	0.09918076211	0.237659562	-2.458647025	6.369150652	MCU		
lipid biosynthetic	2/46	0.02695818018	0.1728569705	0.04345117767	0.237659562	-1.750949632	6.327001332	CG4830;SREBP		
regulation of tran	1/21	0.1103554414	0.1978765693	0.1420953162	0.2575834274	-2.838198257	6.255527568	GluRIIC		
regulation of mito	1/15	0.08011920815	0.1850025352	0.1054380314	0.2456996327	-2.467011858	6.227329154	SREBP		
synaptic transmis	1/14	0.07498176072	0.1779940862	0.09918076211	0.237659562	-2.403874541	6.227261964	GluRIIC		
copper ion home	1/16	0.08522837843	0.1882435489	0.1116526213	0.2466066592	-2.462991185	6.064920774	Grx1		
axonemal dynein	1/19	0.1003877644	0.1978765693	0.1300431629	0.2575834274	-2.540172211	5.839131831	l(2)41Ab		
glutathione meta	2/47	0.0280559328	0.1728569705	0.04510281606	0.237659562	-1.629197014	5.8220254	GstD9;GstT4		
cellular response	1/16	0.08522837843	0.1882435489	0.1116526213	0.2466066592	-2.350572935	5.788099735	Hsc70-1		
response to unfo	1/16	0.08522837843	0.1882435489	0.1116526213	0.2466066592	-2.347314742	5.780076692	Hsc70-1		
organonitrogen c	2/53	0.03500705226	0.1728569705	0.05548383562	0.237659562	-1.71188866	5.738603	CG9458;mal		
cofactor biosynth	1/20	0.10538536	0.1978765693	0.1360898742	0.2575834274	-2.506255218	5.639403943	mal		
regulation of circa	1/19	0.1003877644	0.1978765693	0.1300431629	0.2575834274	-2.453019776	5.638793225	Gabat		
SREBP signaling	1/7	0.03821473511	0.1728569705	0.05416045197	0.237659562	-1.714370263	5.596620187	SREBP		
DNA catabolic pr	1/21	0.1103554414	0.1978765693	0.1420953162	0.2575834274	-2.460616279	5.423318449	DNaseII		
compound eye pi	1/14	0.07498176072	0.1779940862	0.09918076211	0.237659562	-2.091305041	5.417547429	w		
glucose transpor	1/21	0.1103554414	0.1978765693	0.1420953162	0.2575834274	-2.402576722	5.29539643	Tret1-1		
cellular response	1/17	0.09030942576	0.1927612953	0.1178248176	0.2557906296	-2.188984693	5.263443117	Hsc70-1		
hydrogen peroxid	1/13	0.06981588103	0.1779940862	0.09288052583	0.237659562	-1.953962011	5.201239311	Prx2540-1		
translational elon	1/20	0.10538536	0.1978765693	0.1360898742	0.2575834274	-2.308431726	5.194275062	eEF1delta		
medium-term me	1/18	0.09536250357	0.1968225749	0.1239549041	0.2575834274	-2.183877739	5.13226517	MCU		
macromolecule m	1/19	0.1003877644	0.1978765693	0.1300431629	0.2575834274	-2.151807325	4.946391664	rgr		
actomyosin contr	1/21	0.1103554414	0.1978765693	0.1420953162	0.2575834274	-2.213241508	4.878092373	pav		
sodium ion transp	2/63	0.04788244957	0.177495799	0.07441395931	0.237659562	-1.598858319	4.858940406	CG13795;CG13796		
inorganic anion tr	1/26	0.1347983592	0.2153382594	0.1715131007	0.2757235923	-2.410050615	4.829681789	Bmcp		
DNA catabolic pr	1/22	0.1152981585	0.1978765693	0.1480597654	0.2575834274	-2.200486363	4.753565068	DNaseII		
positive regulatio	1/7	0.03821473511	0.1728569705	0.05416045197	0.237659562	-1.449337582	4.731411962	udd		
chaperone media	1/22	0.1152981585	0.1978765693	0.1480597654	0.2575834274	-2.18069458	4.71081019	Hsc70-1		
de novo' posttran	1/22	0.1152981585	0.1978765693	0.1480597654	0.2575834274	-2.173768512	4.695848263	Hsc70-1		
assembly of acto	1/22	0.1152981585	0.1978765693	0.1480597654	0.2575834274	-2.173673509	4.695643034	pav		
calcium ion trans	1/24	0.1251020967	0.2090521879	0.1598667823	0.2689149848	-2.213407756	4.600844922	MCU		
monocarboxylic a	1/17	0.09030942576	0.1927612953	0.1178248176	0.2557906296	-1.912718341	4.599156961	Sln		
inorganic cation t	2/79	0.07138717984	0.1779940862	0.1081892722	0.2466066592	-1.714846806	4.526573045	CG13795;CG13796		
fatty acid elongat	1/21	0.1103554414	0.1978765693	0.1420953162	0.2575834274	-2.043913467	4.5048851	CG9458		
coenzyme biosyn	1/24	0.1251020967	0.2090521879	0.1598667823	0.2689149848	-2.163385672	4.496867763	pr		
organophosphate	1/22	0.1152981585	0.1978765693	0.1480597654	0.2575834274	-2.058363866	4.446547244	mal		
actomyosin contr	1/23	0.1202136608	0.2035617989	0.1539834964	0.2624953563	-2.056574707	4.356821872	pav		
peptide catabolic	1/20	0.10538536	0.1978765693	0.1360898742	0.2575834274	-1.93415803	4.352110009	Psa		
transition metal io	1/21	0.1103554414	0.1978765693	0.1420953162	0.2575834274	-1.923042829	4.238480313	Grx1		

fatty acid elongat	1/21	0.1103554414	0.1978765693	0.1420953162	0.2575834274	-1.914850174	4.2204233	CG9458		
very long-chain fa	1/22	0.1152981585	0.1978765693	0.1480597654	0.2575834274	-1.933815341	4.177493308	CG9458		
fatty acid elongat	1/21	0.1103554414	0.1978765693	0.1420953162	0.2575834274	-1.88863707	4.162648339	CG9458		
fatty acid elongat	1/22	0.1152981585	0.1978765693	0.1480597654	0.2575834274	-1.923295961	4.154768986	CG9458		
negative regulati	1/25	0.1299636138	0.2129726317	0.1657098939	0.2733137212	-2.022656752	4.127232641	Fs		
covalent chromat	1/20	0.10538536	0.1978765693	0.1360898742	0.2575834274	-1.832178521	4.122642699	rgr		
glucose import (C	1/21	0.1103554414	0.1978765693	0.1420953162	0.2575834274	-1.864552076	4.109563836	Tret1-1		
cAMP-mediated s	1/26	0.1347983592	0.2153382594	0.1715131007	0.2757235923	-1.951684305	3.911127049	rk		
regulation of hist	1/27	0.1396064789	0.2202487307	0.1772766701	0.2814267137	-1.947340639	3.834172886	rgr		
ion transport (GC	2/68	0.05487037915	0.177495799	0.08455139395	0.237659562	-1.319489861	3.830190915	MFS15;nAChRalpha3		
very long-chain fa	1/25	0.1299636138	0.2129726317	0.1657098939	0.2733137212	-1.828420234	3.730892879	CG9458		
regulation of hist	1/27	0.1396064789	0.2202487307	0.1772766701	0.2814267137	-1.880134039	3.701847951	rgr		
carbohydrate cat	1/32	0.1632527498	0.2396890084	0.2055091966	0.3034845112	-2.006676686	3.637012531	Mal-A4		
smoothened sign	1/29	0.1491434215	0.2309904211	0.1886859579	0.2938117826	-1.908609043	3.631790753	pav		
hexose metabolic	1/26	0.1347983592	0.2153382594	0.1715131007	0.2757235923	-1.805174784	3.617525594	Gal		
positive regulatio	1/26	0.1347983592	0.2153382594	0.1715131007	0.2757235923	-1.801114641	3.609389169	Fs		
homeostasis of n	1/30	0.1538725327	0.2312640433	0.1943322027	0.2938117826	-1.903691392	3.563007308	rk		
intestinal stem ce	1/30	0.1538725327	0.2312640433	0.1943322027	0.2938117826	-1.878376453	3.51562709	rk		
cilium movement	1/31	0.1585755948	0.2355450355	0.1999398626	0.2987336771	-1.909041631	3.515545714	l(2)41Ab		
fatty acid elongat	1/21	0.1103554414	0.1978765693	0.1420953162	0.2575834274	-1.592185159	3.509253848	CG9458		
anion transport ((	1/28	0.1443881181	0.2263863087	0.1830008678	0.2887094436	-1.789016023	3.462193867	MFS15		
cellular calcium io	1/30	0.1538725327	0.2312640433	0.1943322027	0.2938117826	-1.846591793	3.456137943	MCU		
organic anion tra	1/30	0.1538725327	0.2312640433	0.1943322027	0.2938117826	-1.812963661	3.393198498	w		
inorganic anion tr	1/37	0.186254843	0.2571126638	0.2327899168	0.3231073162	-1.991861468	3.347600901	Bmcp		
adenylate cyclase	1/29	0.1491434215	0.2309904211	0.1886859579	0.2938117826	-1.75467292	3.338873884	rk		
membrane lipid b	1/32	0.1632527498	0.2396890084	0.2055091966	0.3034845112	-1.835331433	3.326456856	CG9458		
protein maturatio	1/37	0.186254843	0.2571126638	0.2327899168	0.3231073162	-1.97571865	3.320470645	CG7791		
regulation of cyto	1/33	0.1679041393	0.243700865	0.2110404617	0.3063101558	-1.800700626	3.213101882	pav		
organonitrogen c	1/30	0.1538725327	0.2312640433	0.1943322027	0.2938117826	-1.697723878	3.177512178	Psa		
divalent metal ion	1/36	0.1817051169	0.2564061095	0.2274083896	0.3226912345	-1.860786178	3.17332919	GluRIIC		
ion transmembra	1/31	0.1585755948	0.2355450355	0.1999398626	0.2987336771	-1.718558998	3.164767402	nAChRalpha3		
purine ribonucleo	1/33	0.1679041393	0.243700865	0.2110404617	0.3063101558	-1.747044829	3.117360513	Adk3		
anterograde trans	2/89	0.08760368325	0.1918218582	0.131009941	0.2575834274	-1.241091868	3.021974596	nAChRalpha3;GluRIIC		
sphingolipid bios	1/37	0.186254843	0.2571126638	0.2327899168	0.3231073162	-1.776349718	2.985403358	CG9458		
cation transport (	1/37	0.186254843	0.2571126638	0.2327899168	0.3231073162	-1.768078809	2.971502942	nAChRalpha3		
oligosaccharide r	1/23	0.1202136608	0.2035617989	0.1539834964	0.2624953563	-1.384265595	2.932545363	Mal-A4		
mitotic spindle as	1/34	0.1725299038	0.2475910422	0.2165339133	0.3124978067	-1.651791884	2.90250343	pav		
regulation of cell	1/38	0.1907794993	0.2619350963	0.2381346356	0.3287293339	-1.737012501	2.87759913	GluRIIC		
negative regulati	1/35	0.1771301833	0.2527588009	0.2219898053	0.3185616415	-1.613709828	2.793122444	pav		
regulation of BMI	1/42	0.2086301522	0.2731549415	0.2591503447	0.3410579665	-1.762389486	2.762003059	GluRIIC		
mitochondrial ger	2/94	0.09608661138	0.1968225749	0.1428101533	0.2575834274	-1.158165037	2.713007729	mRpL45;mIF2		
positive regulatio	1/42	0.2086301522	0.2731549415	0.2591503447	0.3410579665	-1.711962548	2.682974354	GILT2		
glucose homeost	1/36	0.1817051169	0.2564061095	0.2274083896	0.3226912345	-1.562520972	2.664676612	Eno		
protein processin	1/46	0.2260905061	0.2900352957	0.2795965989	0.362334368	-1.761915516	2.619651034	CG7791		
carboxylic acid tr	1/42	0.2086301522	0.2731549415	0.2591503447	0.3410579665	-1.654603849	2.593082249	Bmcp		
calcium ion trans	1/42	0.2086301522	0.2731549415	0.2591503447	0.3410579665	-1.638434209	2.567741314	GluRIIC		
negative regulati	1/40	0.1997541489	0.2698806055	0.2487146347	0.3360293469	-1.561512145	2.515077525	pav		
mitochondrial tra	2/94	0.09608661138	0.1968225749	0.1428101533	0.2575834274	-1.057071274	2.476195053	mRpL45;mIF2		
adenylate cyclase	1/40	0.1997541489	0.2698806055	0.2487146347	0.3360293469	-1.516159958	2.442030211	rk		
lipid homeostasis	1/40	0.1997541489	0.2698806055	0.2487146347	0.3360293469	-1.500580851	2.416937443	SREBP		
negative regulati	3/192	0.09135614842	0.1933705142	0.1525212506	0.2624953563	-0.9792874036	2.343424663	sug;Hr3;CG42741		
carbohydrate hor	1/41	0.2042044139	0.2729890586	0.2539504045	0.3410579665	-1.444751016	2.295180236	Eno		
regulation of mul	1/48	0.2346769711	0.2980397533	0.2896109311	0.3734069873	-1.543265667	2.237033497	rk		
brain developme	1/44	0.2174085836	0.2803136053	0.2694436972	0.3509676877	-1.444359938	2.204059788	bwa		
cellular response	1/43	0.2130314977	0.2760714307	0.2643146959	0.346061509	-1.415281831	2.188471875	Hsc70-1		
proteolysis (GO:(	4/305	0.0900623015	0.1927612953	0.165483774	0.2733137212	-0.8734064491	2.102510827	CG7791;CG18754;CG14990;Psa		
microtubule cytos	1/52	0.251567678	0.3147694099	0.3092312894	0.390769888	-1.446620417	1.99639871	pav		
proteolysis involv	1/52	0.251567678	0.3147694099	0.3092312894	0.390769888	-1.443387606	1.991937292	Ubi-p5E		
translation (GO:0	3/225	0.1298205212	0.2129726317	0.2096634425	0.3063101558	-0.9383889731	1.915817169	mRpL45;mIF2;eEF1delta		
positive regulatio	2/135	0.1725339153	0.2475910422	0.2455328627	0.3360293469	-1.073464044	1.886249637	SREBP;sug		
cellular protein co	1/60	0.2842486216	0.3471112975	0.3468892478	0.4256515408	-1.475164027	1.855617674	l(2)41Ab		
protein targeting	1/52	0.251567678	0.3147694099	0.3092312894	0.390769888	-1.342832067	1.853166301	CG7791		
cellular response	1/64	0.3000547081	0.3629233136	0.3649548405	0.4435336339	-1.437223876	1.730116392	sug		
cell morphogenes	1/58	0.2762134478	0.3405738628	0.3376681858	0.418379118	-1.336936088	1.720077038	SREBP		
neuromuscular sy	1/59	0.2802421464	0.3438720057	0.3422945827	0.4220525437	-1.305592914	1.660846366	GluRIIC		
neuropeptide sig	1/57	0.2721624036	0.3372158562	0.3330098418	0.4146299011	-1.25108825	1.6281116	rk		
mitotic cytokinesi	1/65	0.3039518754	0.3658946747	0.3693940224	0.4467908652	-1.337909063	1.593297031	pav		
cytoskeleton-dep	1/69	0.3193266742	0.379013903	0.3868480861	0.4634878012	-1.356919238	1.548978459	pav		
sensory percepti	1/63	0.2961359182	0.3598972404	0.3604849808	0.4402076207	-1.240219715	1.509268945	Bmcp		
modulation of che	1/70	0.3231174895	0.3817294992	0.3911369523	0.4664262248	-1.271374488	1.436321696	GluRIIC		

regulation of auto	1/72	0.3306364663	0.3852369837	0.3996265094	0.4699311731	-1.228344704	1.359453055	SREBP		
sensory percepti	1/72	0.3306364663	0.3852369837	0.3996265094	0.4699311731	-1.191637765	1.31882817	Bmcp		
regulation of canc	1/68	0.3155148212	0.376247721	0.3825295619	0.4604858233	-1.128057378	1.301270161	pav		
detection of chen	1/73	0.3343648561	0.3878021619	0.4038275962	0.4726829928	-1.180470262	1.293231731	Ir25a		
ubiquitin-depende	2/164	0.2310125824	0.2948602811	0.3202921011	0.4007595747	-0.8758465222	1.283363108	Rpn7;Ubi-p5E		
chemical synapti	2/154	0.2106272943	0.2743555526	0.2945786119	0.3778937748	-0.819725632	1.276857996	nAChRalpha3;GluRIIC		
modification-depe	1/71	0.326887382	0.3843953474	0.3953963606	0.4693022224	-1.110310403	1.241481991	Ubi-p5E		
negative regulati	3/279	0.2022604958	0.2718209837	0.3108489141	0.3908694267	-0.7642325864	1.221395625	sug;Hr3;CG42741		
peripheral nervou	1/79	0.3563069076	0.4058383611	0.428434698	0.49240911	-1.125059336	1.161019405	pav		
mitotic spindle or	1/74	0.3380726647	0.3903202583	0.4079998169	0.4753759334	-1.052138558	1.141038398	pav		
cilium assembly (	1/79	0.3563069076	0.4058383611	0.428434698	0.49240911	-1.010632077	1.042934727	l(2)41Ab		
positive regulatio	2/204	0.3131111475	0.3751426012	0.420278118	0.4874458538	-0.8481119792	0.9848251263	SREBP;sug		
somatic muscle c	1/85	0.3775319622	0.4280942786	0.4520438533	0.5148840302	-1.006831861	0.9807549621	pav		
proteasomal prot	1/94	0.408075771	0.4586338312	0.485672968	0.5507184548	-1.029390273	0.9226449807	Rpn7		
endosomal trans	1/96	0.414658916	0.46397958	0.4928662646	0.5563912498	-1.043307605	0.9184226263	Muted		
regulation of tran	5/623	0.2646392796	0.3295018481	0.4429287432	0.5067743278	-0.6763106451	0.8990789781	CG10654;SREBP;sug;rgr;CG42741		
regulation of nucl	2/246	0.3972605771	0.4484630515	0.5170261188	0.5734700183	-0.8978011819	0.8288166961	CG10654;rgr		
detection of chen	1/98	0.4211694982	0.4691975989	0.4999607608	0.5594274592	-0.9583224249	0.8286804884	Ir25a		
male mating beha	1/101	0.4308010478	0.4778317299	0.5104201641	0.5686259723	-0.9799788263	0.8252488928	w		
regulation of mac	1/113	0.4677645415	0.5055838023	0.5501518454	0.5997363465	-1.018366614	0.7737450002	rgr		
regulation of cellu	2/266	0.4357239237	0.481190768	0.5593353429	0.6045582004	-0.8888047069	0.7383713449	CG10654;rgr		
positive regulatio	3/379	0.3516242786	0.4041292613	0.4981622631	0.5594274592	-0.7048006704	0.7366520673	SREBP;sug;Hr3		
phosphate-conta	1/112	0.4647773272	0.5055838023	0.5469655416	0.5997363465	-0.9053258927	0.6936578511	mal		
courtship behavic	1/109	0.4557156332	0.501089917	0.5372730911	0.5933363701	-0.8666327341	0.6810747716	w		
male courtship be	1/113	0.4677645415	0.5055838023	0.5501518454	0.5997363465	-0.8823972563	0.670436811	w		
protein modificati	1/115	0.4736894914	0.5098183509	0.5564585858	0.6040191486	-0.8755441642	0.654209448	Ubi-p5E		
cellular protein m	1/132	0.521483139	0.547341807	0.6066759417	0.6420653716	-0.9709824597	0.632185645	Ubi-p5E		
DNA metabolic p	1/128	0.5106398246	0.5426883492	0.5953875132	0.6380946344	-0.8892079649	0.597628476	DNaseII		
border follicle cel	1/121	0.4910749721	0.5262997591	0.5748624869	0.6187079308	-0.8247007035	0.5864928906	rk		
negative regulati	1/132	0.521483139	0.547341807	0.6066759417	0.6420653716	-0.898033346	0.5846900574	sug		
sensory percepti	1/130	0.516091579	0.5461969211	0.6010709279	0.6414790575	-0.8834397089	0.5843697923	Ir25a		
positive regulatio	2/305	0.5067500182	0.5408172463	0.6342633297	0.6684767043	-0.7084976502	0.4815923916	SREBP;Hr3		
cellular macromo	1/166	0.6045413121	0.6319073797	0.690942053	0.7222192653	-0.903779173	0.4548587454	eEF1delta		
central nervous s	1/170	0.6133205921	0.6358507363	0.6996063216	0.7253061456	-0.9040317821	0.4419517492	bwa		
regulation of tran	4/630	0.4646077783	0.5055838023	0.6507779119	0.683047891	-0.5579594589	0.4277103608	sug;Hr3;GluRIIC;CG42741		
negative regulati	1/179	0.6323740493	0.6476734215	0.7182383452	0.7356150793	-0.8748522579	0.4009222271	sug		
protein ubiquitina	1/168	0.6089553672	0.6339125543	0.6953044228	0.7238005057	-0.7859127339	0.3898208131	Ubi-p5E		
chitin-based cutic	1/178	0.6303038103	0.6476734215	0.7162255005	0.7356150793	-0.7805490175	0.3602650039	Cpr47Ef		
proteasome-med	1/178	0.6303038103	0.6476734215	0.7162255005	0.7356150793	-0.753531773	0.3477951045	Rpn7		
sensory percepti	1/204	0.6805553048	0.694221074	0.7642429772	0.7795892218	-0.6970398485	0.2682531295	Ir25a		
vesicle-mediated	1/235	0.7316909688	0.7404362792	0.8111497961	0.8241281928	-0.7266718012	0.227010111	Hsc70-1		
nervous system (	1/246	0.7478122849	0.7507680646	0.8254836514	0.832035109	-0.700712841	0.2036294559	pav		
regulation of gen	2/458	0.7256124929	0.7372222928	0.8368486316	0.8401563337	-0.6297000255	0.2019694591	CG10654;rgr		
negative regulati	1/245	0.746387278	0.7507680646	0.8242259995	0.832035109	-0.6468226229	0.1892025215	sug		
cellular protein m	1/391	0.8889363915	0.8889363915	0.9390061869	0.9390061869	-0.6093105294	0.07173388285	Ubi-p5E		

Molecular Function

Term	Overlap	P-value	Adjusted P-value	Old P-value	Old Adjusted P-v	Z-score	Combined Score	Genes	
gamma-aminobu	2/9	0.001071339331	0.05737585913	0.002785653593	0.1430698561	-3.512971309	24.02466874	CG13795;CG13796	
gamma-aminobu	2/10	0.001334322305	0.05737585913	0.003327205957	0.1430698561	-3.531313565	23.37493601	CG13795;CG13796	
anion:cation sym	2/13	0.002287799146	0.07378152247	0.005219691178	0.1683350405	-2.924969976	17.78430006	CG13795;CG13796	
glutamate-gated	1/6	0.03284541638	0.1928852775	0.05036548825	0.2719152235	-4.585082786	15.66238179	GluRIIC	
ligand-gated cati	3/35	0.000956807954	0.05737585913	0.002613876703	0.1430698561	-2.145647054	14.91634062	nAChRalpha3;Ir25a;GluRIIC	
adenylate kinase	1/6	0.03284541638	0.1928852775	0.05036548825	0.2719152235	-4.026924932	13.75574634	Adk3	
transmitter-gated	2/21	0.00598373337	0.1286502675	0.01211726741	0.2605212494	-2.588013463	13.24729194	nAChRalpha3;GluRIIC	
glycerophosphoc	1/6	0.03284541638	0.1928852775	0.05036548825	0.2719152235	-3.832573923	13.09185436	CG11619	
protein-hormone	1/7	0.03821473511	0.1928852775	0.05735510325	0.2719152235	-3.941281703	12.86644852	rk	
sodium:amino ac	2/19	0.004907808451	0.126621458	0.01015173927	0.2605212494	-2.343169401	12.45846249	CG13795;CG13796	
oxaloacetate trar	1/7	0.03821473511	0.1928852775	0.05735510325	0.2719152235	-3.747367321	12.23340841	Bmcp	
ionotropic glutam	2/23	0.007157108519	0.1318952856	0.01423548792	0.262339706	-2.476261849	12.23186491	Ir25a;GluRIIC	
thiosulfate transn	1/7	0.03821473511	0.1928852775	0.05735510325	0.2719152235	-3.715417445	12.12910695	Bmcp	
secondary active	2/25	0.008425684258	0.1358641587	0.01650171509	0.2660901558	-2.474526025	11.81950078	Bmcp;MCU	
transaminase act	1/9	0.04886490887	0.1928852775	0.07118383168	0.2719152235	-3.915364565	11.81929437	Gabat	
ligand-gated calc	1/8	0.04355451225	0.1928852775	0.06429443326	0.2719152235	-3.69312156	11.57329003	GluRIIC	
maltose alpha-glu	1/11	0.05939820044	0.1928852775	0.08481423526	0.2719152235	-3.718871702	10.50020208	Mal-A4	
endodeoxyribonu	1/8	0.04355451225	0.1928852775	0.06429443326	0.2719152235	-3.210361388	10.06044422	DNaseII	
malate transmem	1/10	0.05414608515	0.1928852775	0.07802364949	0.2719152235	-3.340454527	9.740997911	Bmcp	
oxidoreductase a	1/9	0.04886490887	0.1928852775	0.07118383168	0.2719152235	-3.225023115	9.73536357	Dhod	
alpha-glucosidas	1/11	0.05939820044	0.1928852775	0.08481423526	0.2719152235	-3.350622282	9.460453025	Mal-A4	
kainate selective	1/10	0.05414608515	0.1928852775	0.07802364949	0.2719152235	-3.160326493	9.215732027	GluRIIC	
potassium chann	2/33	0.01440828704	0.1928852775	0.02695640804	0.2719152235	-1.999223831	8.476612578	CG42340;GluRIIC	
alpha-amylase ac	1/13	0.06981588103	0.1928852775	0.09824909306	0.2719152235	-3.1571362	8.403961192	Mal-A4	
neurotransmitter	1/11	0.05939820044	0.1928852775	0.08481423526	0.2719152235	-2.93349861	8.282707948	GluRIIC	
excitatory extrace	1/12	0.06462141319	0.1928852775	0.09155593519	0.2719152235	-2.990543133	8.191724008	nAChRalpha3	
succinate transm	1/11	0.05939820044	0.1928852775	0.08481423526	0.2719152235	-2.878630895	8.127789427	Bmcp	
amylase activity (	1/14	0.07498176072	0.1928852775	0.1048940503	0.2719152235	-2.975621421	7.708378195	Mal-A4	
nucleobase-conta	1/8	0.04355451225	0.1928852775	0.06429443326	0.2719152235	-2.424703493	7.5983951	Adk3	
hydrolase activity	2/41	0.02174354883	0.1928852775	0.03942938149	0.2719152235	-1.892942677	7.247013999	CG10505;w	
potassium ion lea	1/12	0.06462141319	0.1928852775	0.09155593519	0.2719152235	-2.64026003	7.232225223	CG42340	
glutamate recept	1/15	0.08011920815	0.1928852775	0.1114911461	0.2719152235	-2.79000675	7.042645666	GluRIIC	
acetylcholine-gat	1/11	0.05939820044	0.1928852775	0.08481423526	0.2719152235	-2.488914948	7.027429824	nAChRalpha3	
AMPA glutamate	1/12	0.06462141319	0.1928852775	0.09155593519	0.2719152235	-2.549524208	6.983680803	Ir25a	
hydrolase activity	2/46	0.02695818018	0.1928852775	0.0481355811	0.2719152235	-1.929817421	6.973334452	CG17110;bwa	
disulfide oxidored	1/15	0.08011920815	0.1928852775	0.1114911461	0.2719152235	-2.590891957	6.540032211	GILT2	
antiporter activity	1/15	0.08011920815	0.1928852775	0.1114911461	0.2719152235	-2.576026748	6.50250886	Bmcp	
glutathione trans	2/41	0.02174354883	0.1928852775	0.03942938149	0.2719152235	-1.686871176	6.458081998	GstD9;GstT4	
leak channel acti	1/12	0.06462141319	0.1928852775	0.09155593519	0.2719152235	-2.31785806	6.349098701	CG42340	
ATPase activity, c	2/48	0.0291714224	0.1928852775	0.05179768182	0.2719152235	-1.766624872	6.244251738	CG10505;w	
ATPase activity, c	2/44	0.02481680305	0.1928852775	0.04457429143	0.2719152235	-1.649963576	6.098651983	CG10505;w	
sulfate transmem	1/16	0.08522837843	0.1928852775	0.1180407172	0.2719152235	-2.45239633	6.038831784	Bmcp	
tRNA binding (GO	1/15	0.08011920815	0.1928852775	0.1114911461	0.2719152235	-2.321798327	5.860775399	mIF2	
aminoacylase ac	1/7	0.03821473511	0.1928852775	0.05735510325	0.2719152235	-1.789333036	5.841338716	CG17110	
endodeoxyribonu	1/16	0.08522837843	0.1928852775	0.1180407172	0.2719152235	-2.306250179	5.678958457	DNaseII	
nucleotide kinase	1/13	0.06981588103	0.1928852775	0.09824909306	0.2719152235	-2.088867303	5.560342866	Adk3	
C4-dicarboxylate	1/16	0.08522837843	0.1928852775	0.1180407172	0.2719152235	-2.236459696	5.50710492	Bmcp	
transmitter-gated	1/16	0.08522837843	0.1928852775	0.1180407172	0.2719152235	-2.220393763	5.467543831	GluRIIC	
deoxyribonucleas	1/10	0.05414608515	0.1928852775	0.07802364949	0.2719152235	-1.794800757	5.233763934	DNaseII	
acetylcholine rec	1/14	0.07498176072	0.1928852775	0.1048940503	0.2719152235	-2.009358892	5.205265079	nAChRalpha3	
metalloaminopep	1/18	0.09536250357	0.2005049568	0.1309986206	0.2775526074	-2.167058975	5.092739901	Psa	
P-P-bond-hydroly	2/60	0.0438601102	0.1928852775	0.07569291182	0.2719152235	-1.624445921	5.079236322	CG10505;w	
translation factor	2/68	0.05487037915	0.1928852775	0.09321606032	0.2719152235	-1.610472694	4.674850532	mIF2;eEF1delta	
fatty acid elongas	1/21	0.1103554414	0.2005049568	0.1500873273	0.2805980467	-2.06267322	4.546232511	CG9458	
phosphotransfera	1/18	0.09536250357	0.2005049568	0.1309986206	0.2775526074	-1.909127727	4.486583457	Adk3	
proteasome bind	1/17	0.09030942576	0.1974562021	0.1245430979	0.2770010282	-1.798851478	4.325362558	Ubi-p5E	
translation elong	1/17	0.09030942576	0.1974562021	0.1245430979	0.2770010282	-1.787125925	4.297168308	eEF1delta	
hydro-lyase activ	1/24	0.1251020967	0.2180833847	0.1687660513	0.3023725086	-2.065570963	4.293547654	Irp-1A	
glucose transmer	1/21	0.1103554414	0.2005049568	0.1500873273	0.2805980467	-1.945554651	4.288097466	Tret1-1	
inorganic anion tr	1/21	0.1103554414	0.2005049568	0.1500873273	0.2805980467	-1.939230538	4.274158812	Bmcp	
fatty acid synthas	1/24	0.1251020967	0.2180833847	0.1687660513	0.3023725086	-2.026803458	4.212964545	CG9458	
organic anion tra	1/19	0.1003877644	0.2005049568	0.137407615	0.2805980467	-1.790322587	4.115441292	w	
hexose transmer	1/21	0.1103554414	0.2005049568	0.1500873273	0.2805980467	-1.854251046	4.086859862	Tret1-1	

cation:sugar sym	1/21	0.1103554414	0.2005049568	0.1500873273	0.2805980467	-1.818838051	4.008807893	Tret1-1	
cholesterol trans	1/13	0.06981588103	0.1928852775	0.09824909306	0.2719152235	-1.438353248	3.828743555	w	
endonuclease ac	1/26	0.1347983592	0.2250665862	0.1809950225	0.3113114387	-1.878020512	3.76350663	DNaseII	
metalloendopepti	1/24	0.1251020967	0.2180833847	0.1687660513	0.3023725086	-1.777562526	3.694886087	CG7791	
RNA polymerase	3/127	0.03378696855	0.1928852775	0.06962641113	0.2719152235	-1.051288337	3.561428575	CG10654;sug;Hr3	
phosphoric dieste	1/16	0.08522837843	0.1928852775	0.1180407172	0.2719152235	-1.38204613	3.403179166	CG11619	
solute:proton sym	1/25	0.1299636138	0.2235374158	0.1749026778	0.309074595	-1.649374234	3.36554938	Tret1-1	
oxidoreductase a	1/27	0.1396064789	0.2279650098	0.1870433976	0.3133584194	-1.693838696	3.335045893	CG8665	
extracellular ligar	1/29	0.1491434215	0.2375247083	0.199009477	0.3209027817	-1.634216176	3.109663145	nAChRalpha3	
fatty acid ligase a	1/26	0.1347983592	0.2250665862	0.1809950225	0.3113114387	-1.545862531	3.097870257	CG4830	
sterol transporter	1/16	0.08522837843	0.1928852775	0.1180407172	0.2719152235	-1.248316821	3.07388133	w	
calcium ion trans	1/29	0.1491434215	0.2375247083	0.199009477	0.3209027817	-1.580390766	3.00724163	MCU	
calcium channel	1/32	0.1632527498	0.2568244479	0.2166365053	0.3450136936	-1.653671262	2.997205849	MCU	
L-amino acid trar	1/35	0.1771301833	0.2688211017	0.2338842499	0.3635068462	-1.687793148	2.921351062	Mal-A4	
sugar:proton sym	1/21	0.1103554414	0.2005049568	0.1500873273	0.2805980467	-1.28768251	2.838115138	Tret1-1	
ligand-gated ion	2/84	0.07936382221	0.1928852775	0.1312458066	0.2775526074	-1.033599813	2.618844926	nAChRalpha3;Ir25a	
metallopeptidase	1/36	0.1817051169	0.2725576754	0.239550596	0.3678812724	-1.49934315	2.556935041	CG7791	
microtubule moto	1/33	0.1679041393	0.2609594454	0.2224274989	0.3499164312	-1.368778865	2.442397078	pav	
monocarboxylic a	1/21	0.1103554414	0.2005049568	0.1500873273	0.2805980467	-0.9893032384	2.180472652	Sln	
aminopeptidase	1/38	0.1907794993	0.2828799472	0.250760686	0.376141029	-1.305874104	2.163359322	Psa	
motor activity (G	1/40	0.1997541489	0.2831679694	0.2618092251	0.3785858269	-1.32480164	2.133815507	pav	
metalloexopeptid	1/39	0.1952792225	0.2831679694	0.2563050065	0.3785858269	-1.249448675	2.04075555	Psa	
RNA polymerase	3/200	0.1001850451	0.2005049568	0.1864966109	0.3133584194	-0.8796780647	2.023907301	CG10654;sug;Hr3	
ATP binding (GO	1/40	0.1997541489	0.2831679694	0.2618092251	0.3785858269	-1.254043933	2.019848338	Hsc70-1	
neuropeptide rec	1/45	0.2217615425	0.2919106018	0.2887386392	0.3879925464	-1.284769839	1.935059443	rk	
sodium channel a	1/45	0.2217615425	0.2919106018	0.2887386392	0.3879925464	-1.255047669	1.89029332	GluRIIC	
transcription regu	3/204	0.1047241908	0.2005049568	0.1939813164	0.3208152541	-0.8125949056	1.833559573	CG10654;sug;Hr3	
translation initiati	1/48	0.2346769711	0.3027332927	0.3044329015	0.3966852959	-1.263934759	1.832130691	mIF2	
carboxylic acid tr	1/42	0.2086301522	0.2919106018	0.2726984882	0.3865725821	-1.123604645	1.760904437	Sln	
ubiquitin protein l	1/44	0.2174085836	0.2919106018	0.2834307186	0.3879925464	-1.151862702	1.757715786	Ubi-p5E	
G-protein couple	1/48	0.2346769711	0.3027332927	0.3044329015	0.3966852959	-1.200207862	1.739755667	rk	
ubiquitin-like prot	1/45	0.2217615425	0.2919106018	0.2887386392	0.3879925464	-1.153057458	1.736680498	Ubi-p5E	
amino acid trans	1/50	0.2431690188	0.3105822121	0.3147071884	0.4059722731	-1.178755476	1.666758506	Mal-A4	
adenyl ribonucle	1/43	0.2130314977	0.2919106018	0.2780840934	0.3879925464	-0.9488968048	1.467293598	Hsc70-1	
transcription fact	2/134	0.1705613025	0.2619334289	0.2641296467	0.3785858269	-0.7593987085	1.3431185	SREBP;CG42741	
nucleoside-tripho	3/230	0.1360867731	0.2250665862	0.2443101777	0.3707766226	-0.5510886593	1.099125698	pav;mIF2;Hsc70-1	
olfactory recepto	1/71	0.326887382	0.3940978718	0.4140184996	0.5126688242	-0.9721148597	1.086960086	Ir25a	
transcriptional re	1/60	0.2842486216	0.3575197724	0.3638946108	0.4616687429	-0.8519856378	1.071717842	CG42741	
ATPase activity (	2/148	0.1984890905	0.2831679694	0.3025476716	0.3966852959	-0.6512279346	1.053049337	pav;Hsc70-1	
ligand-gated cha	1/61	0.2882329948	0.3575197724	0.3686192288	0.4616687429	-0.8053458277	1.001839028	Ir25a	
transcriptional ac	1/76	0.3454269877	0.4088080864	0.4355057157	0.5154150214	-0.8270477788	0.8791302704	SREBP	
transcriptional re	1/73	0.3343648561	0.3993802448	0.4227076323	0.5126688242	-0.7056187672	0.7730212349	CG42741	
sequence-specifi	2/197	0.2987868406	0.3636179475	0.4329065022	0.5154150214	-0.6167230575	0.7450167898	CG10654;CG42741	
endopeptidase a	3/290	0.218057799	0.2919106018	0.3660488194	0.4616687429	-0.4751105449	0.7235910404	CG7791;CG18754;CG14990	
cation channel a	1/86	0.3810014049	0.4427854165	0.4761952272	0.5534160749	-0.740925791	0.7149579841	MCU	
transcriptional ac	1/93	0.4047567374	0.4614570767	0.5029575636	0.572485512	-0.7382835443	0.6677546091	SREBP	
serine-type endo	2/191	0.2864738497	0.3575197724	0.4175173559	0.5126688242	-0.4655021005	0.58192791	CG18754;CG14990	
RNA polymerase	1/92	0.4014192619	0.4614570767	0.4992178462	0.572485512	-0.6179434407	0.5640271691	CG10654	
core promoter pr	1/95	0.4113764637	0.4614570767	0.5103553014	0.572485512	-0.6155589372	0.5467680798	CG10654	
purine ribonucle	1/94	0.408075771	0.4614570767	0.5066699838	0.572485512	-0.6004594853	0.5381932829	Hsc70-1	
G-protein couple	1/102	0.4339761362	0.4826113929	0.5354097213	0.5954125349	-0.6290616371	0.525119098	rk	
sequence-specifi	1/111	0.4617734989	0.4923039782	0.5657881216	0.6031956007	-0.6654691646	0.5141952267	CG10654	
microtubule bindi	1/111	0.4617734989	0.4923039782	0.5657881216	0.6031956007	-0.6461728129	0.4992853068	pav	
ATPase activity,	1/107	0.4495902059	0.4873708955	0.5525341429	0.5989655835	-0.6167879813	0.4930718861	Hsc70-1	
GTPase activity (	1/107	0.4495902059	0.4873708955	0.5525341429	0.5989655835	-0.6091537369	0.4869689278	mIF2	
structural constitu	1/107	0.4495902059	0.4873708955	0.5525341429	0.5989655835	-0.5608976498	0.4483921063	Cpr47Ef	
protein homodim	1/139	0.5398890115	0.5616587297	0.6484042915	0.6745496258	-0.4933590383	0.3041024138	Gal	
transcription regu	1/137	0.5347029924	0.5607860652	0.6430518171	0.6744201985	-0.4846378279	0.3034045271	CG10654	
ion channel activ	1/83	0.3705351612	0.4345366891	0.4642992156	0.5444963529	-0.2830987383	0.2810623911	Ir25a	
serine-type pepti	2/194	0.2926335812	0.3595212569	0.4252369317	0.5126688242	-0.1430144551	0.1757410291	CG18754;CG14990	
mRNA binding (G	1/196	0.6658607939	0.6871683393	0.7719215822	0.7966230729	-0.4288393269	0.1743980825	Irp-1A	
zinc ion binding (	1/211	0.6928865052	0.709383803	0.7966180668	0.8155851636	-0.2742816888	0.1006309527	Psa	
DNA binding (GC	2/448	0.7142275811	0.7254752595	0.8541198778	0.8607926893	-0.2905907063	0.09779935601	CG10654;CG42741	
tubulin binding (G	1/120	0.4882174336	0.5162299093	0.5942223937	0.6283171212	-0.1106882532	0.07936285897	pav	

transition metal i	1/242	0.7420642048	0.7478615814	0.8396614467	0.8528844616	-0.1888608362	0.05634087214	Psa	
RNA binding (GC	1/401	0.8950669492	0.8950669492	0.9536141946	0.9536141946	-0.276173614	0.03061571201	mIF2	

Cellular Component

Term	Overlap	P-value	Adjusted P-value	Old P-value	Old Adjusted P-v	Z-score	Combined Score	Genes
mitochondrion (G	12/465	0.000010652485	0.000500666832	0.0000370467100	0.001741195378	-1.169552712	13.3910479	Prx2540-1;pr;CG3566;Adk3;mRpL45;CG10361;Dhod;mIF2;CG7791;Gabat;MCU;Hsc70-1
mitotic spindle m	1/8	0.04355451225	0.2510401855	0.05915959992	0.3221322743	-3.443827136	10.79206563	pav
female germline	1/10	0.05414608515	0.2510401855	0.0718368042	0.3221322743	-3.673721823	10.71282854	pav
ionotropic glutam	2/25	0.008425684258	0.1390334246	0.01396877378	0.3221322743	-2.011535998	9.60804253	Ir25a;GluRIIC
BLOC-1 complex	1/10	0.05414608515	0.2510401855	0.0718368042	0.3221322743	-3.276070158	9.553248611	Muted
kinetochore micr	1/14	0.07498176072	0.2510401855	0.09669532196	0.3221322743	-3.10016426	8.031007711	pav
AMPA glutamate	1/13	0.06981588103	0.2510401855	0.09054202034	0.3221322743	-2.676795399	7.125345004	Ir25a
acetylcholine-gat	1/11	0.05939820044	0.2510401855	0.07811302365	0.3221322743	-2.521044133	7.1181463	nAChRalpha3
neurotransmitter	1/15	0.08011920815	0.2510401855	0.1028081726	0.3221322743	-2.324549223	5.867719321	GluRIIC
mitochondrial inte	1/14	0.07498176072	0.2510401855	0.09669532196	0.3221322743	-2.187262578	5.666126424	Dhod
polytene chromo	1/15	0.08011920815	0.2510401855	0.1028081726	0.3221322743	-2.113184067	5.334183013	Ubi-p5E
integral compone	7/419	0.00887447391	0.1390334246	0.02146181499	0.3221322743	-1.045196583	4.938110926	CG42340;Tret1-1;Sln;nAChRalpha3;rk;TM4SF;GluRIIC
sensory dendrite	1/15	0.08011920815	0.2510401855	0.1028081726	0.3221322743	-1.792250529	4.524069851	Ir25a
intrinsic compone	1/23	0.1202136608	0.31188098	0.1502853889	0.3825088177	-1.985627059	4.206520372	MCU
mitochondrial env	1/22	0.1152981585	0.31188098	0.1444869854	0.3825088177	-1.918944789	4.145369438	Dhod
cation channel co	1/29	0.1491434215	0.31188098	0.1842824297	0.3825088177	-2.023850251	3.851077127	Ir25a
mitochondrial inn	2/75	0.06520951442	0.2510401855	0.09455084076	0.3221322743	-1.396922669	3.813808277	Bmcp;Dhod
mitochondrial ma	2/69	0.05630881426	0.2510401855	0.08231280772	0.3221322743	-1.318884548	3.794304492	Adk3;Gabat
nuclear speck (G	1/31	0.1585755948	0.31188098	0.1953182961	0.3825088177	-1.996945438	3.677422672	sug
spindle midzone	1/29	0.1491434215	0.31188098	0.1842824297	0.3825088177	-1.926201633	3.66526676	pav
kinesin complex	1/34	0.1725299038	0.31188098	0.2116006226	0.3825088177	-2.035493138	3.576737403	pav
integral compone	1/28	0.1443881181	0.31188098	0.1787095007	0.3825088177	-1.730989093	3.349897232	MCU
spindle microtubu	1/31	0.1585755948	0.31188098	0.1953182961	0.3825088177	-1.808514332	3.330422294	pav
vacuole (GO:000	1/32	0.1632527498	0.31188098	0.2007817024	0.3825088177	-1.681234275	3.047162589	Gal
integral compone	1/33	0.1679041393	0.31188098	0.2062090676	0.3825088177	-1.657257319	2.957147088	MCU
mitotic spindle (G	1/33	0.1679041393	0.31188098	0.2062090676	0.3825088177	-1.579221946	2.817903727	pav
integral compone	1/52	0.251567678	0.4222743166	0.3027797667	0.5082374656	-1.538073758	2.122608274	CG9458
mitochondrial larg	1/50	0.2431690188	0.4222743166	0.2931781845	0.5082374656	-1.34262687	1.898472417	mRpL45
histone methyltra	1/57	0.2721624036	0.4410907921	0.3262327301	0.5287220108	-1.321725036	1.720035227	rgr
nuclear body (GO	1/62	0.292195387	0.4430059093	0.348918588	0.5290056011	-1.320524301	1.624684052	sug
cytoplasmic vesi	1/60	0.2842486216	0.4430059093	0.3399347203	0.5290056011	-1.144892318	1.440166912	w
microtubule (GO	1/85	0.3775319622	0.5370748277	0.4440650624	0.6280834128	-1.114173839	1.085316788	pav
polymeric cytosk	1/90	0.3946885787	0.5370748277	0.4628848627	0.6280834128	-1.111172984	1.033011113	pav
microtubule orga	1/111	0.4617734989	0.5601162661	0.5353899837	0.6490750503	-1.183299026	0.9143124032	pav
microbody (GO:0	1/94	0.408075771	0.5370748277	0.477493706	0.6280834128	-0.9861880166	0.8839226945	CG12355
spindle (GO:000	1/95	0.4113764637	0.5370748277	0.4810851672	0.6280834128	-0.9878359386	0.8774418284	pav
peroxisome (GO	1/94	0.408075771	0.5370748277	0.477493706	0.6280834128	-0.8981300641	0.8049961396	CG12355
centrosome (GO	1/108	0.4526614112	0.5601162661	0.5256471276	0.6490750503	-0.9200798992	0.729265329	pav
microtubule cytos	1/112	0.4647773272	0.5601162661	0.5385941907	0.6490750503	-0.8757521385	0.6709985337	pav
chromosomal reg	1/132	0.521483139	0.6127426883	0.5983500476	0.7030613059	-0.802210578	0.5223019289	Ubi-p5E
nucleoplasm part	1/157	0.5840591404	0.6535899904	0.6625446518	0.7414190151	-0.8181203507	0.4399467004	rgr
nucleolus (GO:0	1/157	0.5840591404	0.6535899904	0.6625446518	0.7414190151	-0.7752851271	0.4169119289	udd
polytene chromo	1/196	0.6658607939	0.6954546069	0.7432711228	0.7939486994	-0.6839953991	0.2781635885	Ubi-p5E
dendrite (GO:003	1/191	0.6563385909	0.6954546069	0.7340806498	0.7939486994	-0.571838425	0.240788854	Ir25a
nucleus (GO:000	9/1776	0.6614838377	0.6954546069	0.8996709494	0.9194502877	-0.4134964727	0.170885575	CG10654;SREBP;sug;pav;Ubi-p5E;Hr3;rgr;CG42741;Hsc70-1
cytosol (GO:000	2/573	0.8311494539	0.8492179203	0.9129256504	0.9194502877	-0.4413525894	0.08162624243	Prx2540-1;Hsc70-1
nuclear lumen (G	1/358	0.8660759166	0.8660759166	0.9194502877	0.9194502877	-0.5446034084	0.07830455434	udd
