# Supplementary material for: Drosophila embryos allocate lipid droplets to specific lineages to ensure punctual development and redox homeostasis
Source: PLoS Genet. 2023 Aug 14;19(8):e1010875. doi: 10.1371/journal.pgen.1010875 (PMC10449164; doi:10.1371/journal.pgen.1010875)
Supplement: Attachment — Submitted filename: Response to Reviewers.docx [file pgen.1010875.s018.docx]

**Reviewer #1**

**Comment**

This study dissects how the asymmetric inheritance of lipid droplets (LD) in the developing Drosophila embryo impacts organismal homeostasis and development. They use specific mutants (Jabba and Klar KO) to affect the inheritance of LDs into cells during embryo cellularization. LD-allocation mutant embryos appear to display defects in LD turnover, development, and delayed hatching. Embryos deficient in perilipin dPLIN2 also display fewer LDs and similar developmental delays. Transcriptomic and proteomic analysis of control and mutant embryos revealed changes in several genes/pathways, including redox homeostasis, sugar metabolism, and mitochondrial metabolism. Of these, proteins involved in glutathione (GSH) redox homeostasis were further investigated. Genetic perturbation of GSTT4 in the Jabba and dPLIN2 mutant backgrounds led to further developmental defects, suggesting an importance of this particular for GST enzyme in the absence of LD homeostasis. Consistent with this, LD-defective mutants display increased lipid peroxidation. LD-allocation mutants also display increased dependence on the ATGL triglyceride lipase.

This is an interesting study that dissects the role of LDs and LD inheritance in Drosophila development. It nicely uses a variety of developed genetic tools to profile how blocking proper LD allocation can impact embryo development. It also implies a protective role for LDs in redox homeostasis in Drosophila. The experiments are generally well conducted. Some additional work dissecting how redox proteins, and in particular ATGL, contribute to organismal development in the absence of proper LD allocation will strengthen the current study.

**Response**

We thank the reviewer for the kind words and constructive feedback.

**Comment**

Specific concerns:

1) In Figure 3A, it is noted that while early stage larva had similar TAG levels, later stage Jabba and Klar KO embryos had elevated TAG levels compared to wildtype. This is interpreted as a defect in LD turnover in these mutants. All these results are compared to sterol levels as a method of normalization. It is generally more common to standardize TAG levels to total protein levels, rather than sterol levels. Are these trends also observed if normalized to protein?

**Response**

We have updated Figure 3A to be controlled against both sterol and protein levels. We are continuing to include sterol levels to serve as a loading reference for the TLC plates. This is now explicitly described in the methods.

**Comment**

2) The end of the manuscript indicates that GST redox enzymes and the triglyceride lipase dATGL are important for organismal survival in the LD-allocation mutants. The redox enzymes are proposed to be important to protect against lipid/protein oxidation, but the importance of dATGL is less clear. It is proposed that it is needed to consume mis-allocated LDs. However, it seems that dATGL may be needed to supply fatty acids for oxidative metabolism and bioenergetics. Investigating this seems important, since mitochondrial metabolism genes/proteins were also upregulated in the LD-allocation mutants. Does perturbation of mitochondrial fatty acid oxidation also perturb survival in the LD-allocation mutants?

**Response**

We have previously attempted to follow this line of inquiry. Zygotic knock down of the fatty acid import machinery CTP1/Whd did not affect viability in wild type or mutant conditions. However, there is a large maternal supply of CPT1/Whd protein. To further respond to this comment, we attempted to make a *whd-/-;klar-/-* double mutant line using the *klar^YG3^* and *whd^1^* alleles. The double mutant is inviable, supporting a link between the mitochondria and LD allocation mutants as the reviewer suggests. We mention these results in the discussion (lines 675-681).

**Comment**

3) Related to point 2, if LD-allocation mutants have increased lipid peroxidation and ROS associated damage, what is the major source of the ROS? It is possibly from alterations in mitochondrial metabolism? Some discussion of this, or some profiling of how mitochondrial metabolism is altered in the LD mutants would mechanistically add to the study.

**Response**

We indeed suspect mitochondrial metabolism is involved in the ROS production, based on our proteomics and transcriptomics data. This has been added to the text (lines 668-681).

**Comment**

4) The text is a bit long, and clarity would be improved by shortening the Introduction and Results sections

**Response**

We agree that the text is lengthy, but we have left it as is to make the story relatively self-contained for newcomers to Drosophila embryos or lipid droplets.

**Reviewer #2**

**Comment**

Here Kilwein, Dao, and Welte use the fruit fly Drosophila melanogaster as a model to investigate how the distribution of embryonic lipid droplets alters development and viability. As a first step towards this goal, the authors use mutations in the genes Jabba and klar to determine how disruption of lipid droplet movement alters embryonic development. In these mutant backgrounds, lipid droplets fail to redistribute to peripheral cells and instead move to the yolk cell with glycogen granules. These mutant studies reveal that the mislocalization of lipid droplets results in decreased embryonic lipid consumption as well as developmental delays. A complementary analysis of dPLIN2, Jabba and klar mutants using a combined RNAseq/proteomics approach reveals a link between decreased lipid consumption and glutathione metabolism. Notably, Jabba and PLIN2 mutants are sensitive to loss of GSTT4 and lipid deprived embryos display elevated levels of peroxidated lipids.

Overall, I found the study to be important and potentially interesting. The topic of embryonic lipid distribution is understudied and the authors make novel observations that could serve as the foundation of many future studies. Moreover, the experiments seem well executed, logical, and appropriately interpreted.

**Response**

We thank the reviewer for their positive and thoughtful assessment of our work as well as for the suggested experiments which improved our understanding of GSTT4’s role in embryogenesis.

**Comment**

However, I have a few concerns that should be addressed:

1. Lines 279-284: It would be nice to show how lipid droplet levels change in embryos from the LpR1; Lpr2/+ cross. Either Nile Red or TLC is fine.

**Response**

We have incorporated this suggestion. The Nile red intensity quantification is now in Fig. 4F. Embryos from LpR1; Lpr2/+have ~70% of the Nile red intensity of wild type.

**Comment**

2. Lines 443-450: I’m a bit surprised that RNAi depletion of only GSTT4 is sufficient to induce a synthetic phenotype with Jabba and PLIN2. I’m a bit worrited that the RNAi vector targeting GSTT4 would also have off-target effects on other GSTs. The authors should provide evidence that the RNAi TRiP line doesn’t have off target effects. For example, does a TRiP line against a different GST result in a similar sensitivity? I’d note that BDSC 77777 is a viable Trojan insertion in GSTT4, which would allow for a confirmatory genetic experiment.

**Response**

Our attempt to cross the *GSTT4* (BDSC 77777) line into a *Jabba-/-* background was unsuccessful. The double mutant is seemingly inviable. We noted that the *GSTT4* (BDSC 77777) line is sickly. Excitingly, we found that that ~80% of the *GSTT4* (BDSC 77777) line’s embryos fail to hatch. Further, the majority of the embryos that fail to hatch are stalled in stage 17, like our LD-deprived embryos. The above data and comments have been added to Figure 7 and discussed in the text (lines 457-469).

**Comment**

3. Lines 389-411 – The text completely lacks references. In particular, many of the genes involved in glutathione metabolism have been previously studied in the fly and the text should reference the appropriate literature.

**Response**

We apologize for this oversight. We have incorporated such references into the relevant paragraph (now lines 392-401).

**Comment**

4. Line 412-13 – Again, please cite the appropriate Drosophila literature. For example, Gclc mutations are known to alter glutathione levels (10.1074/jbc.M308035200).

**Response**

We have incorporated this suggestion (line 416).

**Comment**

5. In general, data presented in the figures lack an adequate explanation of the statistical test used and what error bars represent. Please include this information in the relevant figure legends. Also, the methods explain that a student’s t-test was used throughout. This test is not appropriate for all of the experiments. For example, Figure 7A is clearly making multiple comparisons and a t-test is not appropriate for this analysis. Also, please check to make sure a parametric test is appropriate for each dataset.

**Response**

We thank the reviewer for pointing out the lack of clarity. We have added information on the statistical tests performed and what errors represent to the figure legends.

For the example of figure 7A, the statistics shown are independent 1-to-1 comparisons between the respective mutant genotypes and the same wild type data set done by DEseq2. We chose to represent them on the same graphs as the wild-type data is the same, to emphasize commonalities in our LD-deprived genotypes, and highlight instances when the three LD-deprived genotypes do not agree, as in Fig 8a.

**Comment**

6. Also regarding the figure, some data is present as histograms with no data point, some as histograms with data points, and some as a scatter plot. Please be consistent.

**Response**

We deliberately used different graph styles for different types of data (bar graphs for mRNA levels, scatter plots for immunofluorescence intensity, etc.) and feel that helps the reader make sense of the data. However, if this is not acceptable, we would be happy to convert the various graphs to a single style.

**Comment**

7. In Figure 3C, the data are presented as normalized TAG. The methods state that TAG levels were first normalized to protein using a Bradford assay. If so, the legend and y-axis label should state this.

**Response**

Legend and y-axis label have been updated.

**Comment**

8. Please acknowledge FlyBase and the appropriate grant number in the acknowledgement section – much of this analysis is made possible by FlyBase. Also, the acknowledgement of the BDSC should include the appropriate grant number.

**Response**

We have incorporated these suggestions into the acknowledgment section.

**Reviewer #3**

**Comment**

Kilwein et al describe a novel role for redox biochemistry in lipid partitioning and overall fitness of the developing embryo. Lipid droplet transport via Jabba and klar is fundamental and essential for the embryonic developmental rate and fuels the efficient progress of insect (and probably many species) embryogenesis. This seemingly conserved phenomenon is news to me and integrates cytoskeletal mechanics, energy and redox homeostasis, careful developmental staging and microscopy, and omics to elucidate an antioxidant mechanism that compensates for problems in lipid metabolism during development.

This manuscript combines genetics, genomics, proteomics, and biochemistry to produce a major advance in two fields- developmental biology and metabolism. It’s summarized well in the Discussion: “Here we provide the first evidence that LD allocation amongst embryonic lineages impacts subsequent development.”

**Response**

We thank Review 3 for their thorough and positive feedback which has improved that manuscript.

**Comment**

Major concerns: none. It’s possible to improve the manuscript, but only incrementally.

**Response**

Thank you!

**Comment**

Minor concerns:
line no. 122: Are these mechanisms completely separate? Consider disparate or another term. Combining the two alleles probably isn’t feasible but could provide information on whether they are independent or whether one is upstream or downstream of the other. Their model suggests that the mechanisms are likely to interact.

**Response**

We have changed the wording to “disparate”. *Jabba^-/-^; klar^-/-^* double mutants are nonviable, which supports disparate mechanisms in addition to the data presented.

**Comment**

472+: The text describes peroxidation of a single (artificial) lipid and this may or may not represent peroxidation of endogenous lipids. C11 is rare in vivo and is exogenously supplied and is unlikely to be in the same subcellular location as the lipids in the droplet. Your genetics experiments certainly support a role for redox balance in these mutants, but it’s more accurate to use “increased levels of BODIPY-C11 lipid peroxidation” as in line 493 instead of implying that the cellular lipids themselves have undergone peroxidation, which may not be true. This would require lipidomics and you haven’t done that.

**Response**

We agree with the reviewer’s point and shifted the language of the text to indicate Bodipy-C11 is exogenous.

**Comment**

484: “This oxidative burden does not seem to compromise mitochondrial function” has not been shown. The authors only assayed mitochondrial membrane potential which is not a measure of its function. Mitochondrial function could be measured by the ATP produced or oxygen consumption rate, which would presumably be different if fatty acid oxidation is reduced and glycogen catabolism increased, as they posit. In this case, I would recommend being more conservative with the conclusions drawn.

**Response**

We have reworded this section (lines 501-508).

**Comment**

The Discussion is good and addresses the relevant fields and implications of the work.
The Methods also seem to be appropriate and complete.

**Response**

Thank you!

**Comment**

Check that genes and mRNAs are in italics. Sometimes -/- is superscript and other times not.

**Response**

We have changed them all to superscript.

**Comment**

Figures
All figs: should add n to legends. Is n=3 only 3 embryos or 3 groups of embryos. 3 is a small n for Drosophila, at least for experiments that aren’t costly.

**Response**

N values have been added as requested.

**Comment**

Graphical abstract: suggest changing the color of mesoderm to differ from the redox stress color.

**Response**

We have adopted this suggestion.

**Comment**

One nucleus is outlined in black and the others aren’t. Do you want to label this as a nucleus, or is it even needed for the purpose of this graphic?

**Response**

We have now outlined all nuclei the same way.

**Comment**

What is the second cell type for ectoderm? If this is meant to represent “more than one kind of ectoderm,” that is also true for meso and endoderm origin cells. May want to consider additional labeling or a legend for the graphical abstract.

**Response**

Additional cell types have been added for the meso and endoderm to indicate that each germ layer gives rise to multiple cell types. As this is only meant to illustrate diversity, we opted not to add more labeling or a legend. However, if desired, we would be happy to do so.

**Comment**

Fig 1: font is quite small.

**Response**

We have increased the font to improve readability.

**Comment**

1A is singular for all species except flies; suggest “fly”

**Response**

Incorporated as suggested.

**Comment**

spelling error in 1A “asymmetrically” and 1C “microtubules” and “strores”

**Response**

Sorry about this. This has been corrected.

**Comment**

The descriptions for 1C seem like they should be incorporated into the legend.

**Response**

Implemented as suggested.

**Comment**

How do the stages in 1C correspond to the numbered embryogenesis stages used elsewhere in the manuscript and literature?

**Response**

The legend now states that these correspond to stages 1-3 (left) and stage 5 (right). Is this sufficient?

**Comment**

Fig 2A: microtubules should connect to the nucleus.
Jabba -/- LDs look larger than controls (and klar mutants) in the micrographs, not smaller as shown in the 2A cartoon.

**Response**

We connected microtubules to the nucleus and have resized the *Jabba^-/-^* LDs in the cartoon. We believe that LDs in *Jabba^-/-^* are close to wild-type size; however, they often cluster together, which by light microscopy (panels 2B) can make them appear fused into a larger structure.

**Comment**

Fig 3A graph panels should be the same size.
3C: are there significant differences here? Error bars may be missing from some columns.
3D-3E: align these so the Y axes are the same level and make fonts the same size for both

**Response**

We have incorporated these suggestions for Figure 3.

**Comment**

Fig 5A: it looks like a small space between some of the dorsal appendages and vitelline membrane
Fig 5D: colors for down-regulated and the genes don’t match
5E: log base 2 – should make it a subscript
Fig 5F: gene track seems like it belongs at the top next to the gene name. Italicize the gene name.

**Response**

We have incorporated these suggestions for Figure 5.

**Comment**

Fig 6. Differentially expressed is a term that typically refers to RNA levels, including in the previous fig.
Consider the term differentially abundant or differentially present, which better represent the observation made and distinguish the two approaches more clearly for the reader. 6C says “genes which are differentially expressed” and “scaled to the number of genes matching the term” which may be confusing if you used proteins.

**Response**

We have incorporated this suggestion.

**Comment**

Some fonts are very small here. Are all of these graphs needed? The GO and subsequent biochemical analyses make the role for GSH clear.

**Response**

We explicitly reference the genes shown in the graphs in the main text so we would prefer to keep them included. We have increased the font size of the protein name to try to make the figure more accessible.

**Comment**

Fig 7. Please make graph and font sizes consistent, also the colors that denote PLIN2 and Klar are changing throughout the fig, would be better if this were consistent too.

**Response**

We have incorporated this suggestion.

**Comment**

Fig 8. Oregon R isn’t the best paternal control genotype as it’s not matched to the RNAi. These transgenic flies are typically made in a different white-eyed genetic background. It *IS* reassuring that there is no difference between OR and ATGL RNAi in the wild-type maternal background. And there is some pretty elaborate genetics and a lot of data to support their model so that repeating the analyses with another control may not be practical.

**Response**

We agree that the suggested paternal control would be slightly better for indicated RNAi experiments but choose to use OrR as the paternal outcross genotype for all the experiments for consistency. For example, all of the paternal genotypes in the mRNAseq experiments are OrR.

**Comment**

8D. Make graphs the same size; genotypes and mRNAs should be italicized in all text and figs

**Response**

We have incorporated this suggestion.

**Comment**

Video 4- the labels for genotypes flash quickly and I missed them the first time.
Consider adding this info to the video legend in lines 1050-1051 “Wild Type on the left, klar -/- on the right”

**Response**

We have incorporated this suggestion.
